# MEK inhibition enhances the response to tyrosine kinase inhibitors in acute myeloid leukemia

**DOI:** 10.1038/s41598-019-54901-9

**Published:** 2019-12-09

**Authors:** María Luz Morales, Alicia Arenas, Alejandra Ortiz-Ruiz, Alejandra Leivas, Inmaculada Rapado, Alba Rodríguez-García, Nerea Castro, Ivana Zagorac, Miguel Quintela-Fandino, Gonzalo Gómez-López, Miguel Gallardo, Rosa Ayala, María Linares, Joaquín Martínez-López

**Affiliations:** 1 H12O-CNIO Haematological Malignancies Clinical Research Unit, Hospital 12 de Octubre – Centro Nacional de Investigaciones Oncológicas, Madrid, Spain; 2 Servicio de Hematología, Hospital 12 de Octubre, Madrid, Spain; 3 Centro de Investigación Biomédica en Red Cáncer (CIBERONC), ISCIII, Madrid, Spain; 4 Breast Cancer Clinical Research Unit, Centro Nacional de Investigaciones Oncológicas, Madrid, Spain; 5 Bioinformatics Unit, Centro Nacional de Investigaciones Oncológicas, Madrid, Spain; 6 Universidad Complutense de Madrid, Madrid, Spain

**Keywords:** Translational research, Targeted therapies, Cancer therapeutic resistance

## Abstract

FMS-like tyrosine kinase 3 (FLT3) is a key driver of acute myeloid leukemia (AML). Several tyrosine kinase inhibitors (TKIs) targeting FLT3 have been evaluated clinically, but their effects are limited when used in monotherapy due to the emergence of drug-resistance. Thus, a better understanding of drug-resistance pathways could be a good strategy to explore and evaluate new combinational therapies for AML. Here, we used phosphoproteomics to identify differentially-phosphorylated proteins in patients with AML and TKI resistance. We then studied resistance mechanisms *in vitro* and evaluated the efficacy and safety of rational combinational therapy *in vitro*, *ex vivo* and *in vivo* in mice. Proteomic and immunohistochemical studies showed the sustained activation of ERK1/2 in bone marrow samples of patients with AML after developing resistance to FLT3 inhibitors, which was identified as a common resistance pathway. We examined the concomitant inhibition of MEK-ERK1/2 and FLT3 as a strategy to overcome drug-resistance, finding that the MEK inhibitor trametinib remained potent in TKI-resistant cells and exerted strong synergy when combined with the TKI midostaurin in cells with mutated and wild-type *FLT3*. Importantly, this combination was not toxic to CD34+ cells from healthy donors, but produced survival improvements *in vivo* when compared with single therapy groups. Thus, our data point to trametinib plus midostaurin as a potentially beneficial therapy in patients with AML.

## Introduction

Activating mutations in FMS-like tyrosine kinase 3 (FLT3) are present in up to 35% of patients with acute myeloid leukemia (AML). FLT3 is a member of the class III receptor tyrosine kinase family and plays important roles in modulating the proliferation and differentiation of hematopoietic stem/progenitor cells by activating downstream mitogenic signaling pathways such as Ras/MAPK, JAK/Stat5, and PI3K-Akt^1,2^. Two major classes of activating mutations have been identified in *FLT3*: internal tandem duplications (ITDs) of 3 to 400 bp within the juxtamembrane domain (JMD), which is the most prevalent form of mutant *FLT3*^3^; and point mutations in the activation loop^4^ of the tyrosine kinase domain (TKD). Less common mutations include activating point mutations in a 16-amino-acid stretch of the *FLT3* JMD^3,5,6^ or ITDs in the TKD-1^7^. All of these mutations ultimately drive constitutive activation of the FLT3 receptor and activate its downstream oncogenic pathways^5,7,8^.

To date, more than 20 FLT3 inhibitors have been developed, and eight of them have been evaluated in clinical trials^9–11^. For example, the tyrosine kinase inhibitor (TKI) sorafenib, which is currently approved for the treatment of renal cell carcinoma, hepatocellular carcinoma, and radioactive iodine-refractory thyroid cancer, produces a high response rate in *FLT3*-ITD-positive relapsed/refractory AML^12^. Moreover, a large phase III study demonstrated the efficacy of sorafenib in the upfront setting of young patients with AML, with superior event-free survival, relapse-free survival, and leukemia-free survival in the sorafenib arm^13^. Another relevant TKI is midostaurin, which inhibits FLT3 and exhibits antiproliferative effects in mutant and wild-type (WT) *FLT3* cells^14^. The initial results of a phase III international study demonstrated a survival benefit in the midostaurin arm^15^. Indeed, midostaurin is the only FLT3 inhibitor approved in combination with intensive chemotherapy for adult patients with AML exhibiting activating *FLT3* mutations^10^.

Despite these encouraging results, all of the TKIs tested so far have failed to show an efficient response in AML when used as a single drug^8,9^, and either did not generate a sufficient initial response, or failed to sustain therapeutic benefits because of secondary resistance^11^. Among the possible mechanisms for these failures is the existence of independent, alternative survival pathways, such as casein kinase 2 alpha, CD47, CD123, PIM, PI3K/AKT/mTORC, JAK/STAT, and MAPK^1,3,16–20^. Accordingly, the characterization of resistance mechanisms is important for the design of new drugs targeting downstream or parallel FLT3 pathways^2,9^.

Resistance to TKIs *via* ERK1/2 activation has been reported in different cancers, both *in vitro* and *in vivo*^3,18^. These observations suggest that ERK inhibition could overcome TKI resistance. Moreover, concomitant blockade of ERK1/2 and FLT3 signaling pathways may provide clinical benefit for the treatment of a subset of patients with AML, as previously suggested^16,21,22^.

In recent years, there has been great interest in developing clinically effective small-molecule inhibitors of MEK, the kinase upstream of ERK1/2, to inhibit the Ras-Raf-MEK-ERK1/2 pathway in cancer^23^. In the setting of AML, the MEK inhibitor trametinib has produced good results in preclinical models^24^, and it has been approved by the US Food and Drug Administration (FDA) for the treatment of several types of cancer. Trametinib was recently evaluated in a phase I/II study in patients with relapsed/refractory leukemia, showing activity in *RAS*-mutated AML^25^. Similarly, it has produced encouraging results in combination with TKIs in preclinical xenograft models of renal cell carcinoma^26^.

The present study provides evidence for the potential therapeutic benefit of the combination of the FLT3 inhibitor midostaurin and the MEK inhibitor trametinib, not only in patients with mutated *FLT3*, but also in those without the mutation. The MEK inhibitor trametinib remained potent in TKI-resistant cells and exerted strong synergy when combined with midostaurin in mutated and wild-type *FLT3* blast cells. Finally, the combination demonstrated statistically significant survival improvements in *in vivo* models when compared with vehicle and monotherapy groups.

## Methods

### Cell cultures, patients and healthy donors, and drugs

Human MOLM-13 (*FLT3*^ITD/WT^) and OCI-AML3 (*FLT3*^WT/WT^) AML cell lines were obtained from the DSMZ culture collection (Braunschweig, Germany). MOLM-13 TKI-resistant cells (MOLM-13R) were produced from parental MOLM-13 after sustained and increasing exposure to sorafenib. For experiments of sorafenib-resistant cell enrichment (n = 3), 5,6-carboxyfluorescein diacetate succinimidyl ester (CFDA-SE)-stained MOLM-13 cells were treated with 5 µM sorafenib over 48 hours, and then proliferative and viable cells (CFDA-SE+, Annexin V-) were sorted on the FACSAria™ Fusion sorter platform (BD Biosciences, San Jose, CA, USA).

For experiments on primary cells, mononuclear cells were obtained from patients with AML or healthy donors by standard density gradient centrifugation on Ficoll cushions. The main characteristics of the patients are summarized in Table 1. The study was approved by the *Comité Ético de Investigación Clínica* of the *Instituto de Investigación Biomédica* of the *Hospital 12 de Octubre*, and all patients and donors provided written informed consent in accordance with the Declaration of Helsinki.

**Table 1:** AML patients’ main characteristics

Demographic data			Clinical features								Performed analysis
P (#)	Sex (M/F)	Age (y)	AML subtype (FAB)	Moment	Sample type	Blasts (%)	Cytogenetics (FISH)	FLT3 status	Other mutations	TKI treatment	Methods
1	M	71	M1	Diagnosis	BM	80	46, XY	L576P	No	Sorafenib	A
				d + 5	PBMC	67 (PB)					B
				d + 15	PBMC	40 (PB)					C
				d + 188	PBMC	81 (PB)					C
				d + 191	PBMC	81 (PB)					B
				d + 195	PBMC	81 (PB)					B
2	M	63	M1	Diagnosis	BM clot	93	47, XY; der (2;8), +5, −7, +8	L576Q	No	Sorafenib	D
				Relapse	BM clot	35					D
3	F	36	M2	Diagnosis	BM clot	77	46, XX	ITD	*NPM1, DNMT3A, CBL*	Midostaurin	D
				Relapse	BM clot	39					D
4	M	66	M1	Relapse	BMMC	94	46, XY; +13,−21	WT	bi*CEBPA*	No	E
5	F	59	M5	Diagnosis	BMMC	71	46, XX; del (8p)	WT	*NPM1*	No	E
6	M	76	M1	Relapse	BMMC	40	46, XY; −21	WT	No	No	E
7	M	75	M1	Diagnosis	BMMC	90	46, XY; del (8p)	WT	*CEBPA*	No	E
8	F	57	M5	Diagnosis	BMMC	78	47,XX; + 8, inv (16) (p13q22)	WT	*CBFB/ MYH11*	No	E

P (#), patient number; M, male; F, female; y, years; d + , day; PBMC, peripheral blood mononuclear cells; BM, bone marrow; BMMC, bone marrow mononuclear cells; PB, peripheral blood; ITD, internal tandem duplication; WT, wildtype; biCEBPA, biallelic CEBPA mutations; TKI, tyrosine kinase inhibitor; A, whole exome sequencing; B, western blot; C, liquid chromatography tandem-mass spectrometry analysis; D, immunohistochemistry analysis; E, drug sensitivity assay.

Sorafenib and trametinib were purchased from Selleck Chemicals (Houston, TX, USA). Midostaurin was purchased from MedChemExpress (Sollentuna, Sweden). For details see *Supplementary Information*.

### Whole exome sequencing

Exonic sequences from genomic DNA samples of patient #1 (bone marrow) at diagnosis were isolated, captured, amplified, and purified following the Ion TargetSeq™ Exome Enrichment manual (MAN0006730, Life Technologies S.A., Madrid, Spain). Sequencing was carried out on the Ion Proton™ System (ThermoFisher Scientific, Waltham, MA, USA). The Ion PGM system (ThermoFisher Scientific) was used for results validation. For details see *Supplementary Information*.

### Liquid chromatography tandem-mass spectrometry analysis

Samples were lysed and digested following the standard filter-aided sample preparation method. Phosphopeptides were enriched in TiO_2_ micro-columns and analyzed by liquid chromatography tandem-mass spectrometry (LC-MS/MS) using an LTQ Orbitrap Velos mass spectrometer (ThermoFisher Scientific). The MaxQuant and MaxLFQ platforms were used for analysis and quantification, respectively. Further data analysis was performed with Perseus. For details see *Supplementary Information*.

### Histopathology and immunohistochemistry

Paraffin-embedded tissues from patients or mice were used for phospho-ERK1/2 (ref. 4370) or human CD45^27^(ref. 13917) detection, respectively (both from Cell Signaling Technology Inc., Danvers, MA, USA). Slides were counterstained with Carazzi’s hematoxylin solution (PanReac AppliChem, Darmstadt, Germany). For details see *Supplementary Information*.

### Immunoblotting assays

Whole cell lysates from drug-treated cultured cells or AML patients’ mononuclear cells were analyzed by western blotting (n = 3). Densitometry analysis of protein expression was corrected for housekeeping protein expression and normalized to control samples. For details see *Supplementary Information*.

### Drug sensitivity assay

Cell viability after monotherapy and combinational treatment was determined after 48 or 72 hours of exposure to drugs or dimethyl sulfoxide (DMSO) in cell lines (n = 3–6) or primary cells (n = 5), respectively, using the Cell Counting Kit-8 reagent from Sigma-Aldrich (St. Louis, MO, USA)^28–30^. For monotherapy treatments, the half maximal inhibitory concentration (IC_50_) values were determined according to a nonlinear regression program (GraphPad Prism 5.01; GraphPad Software Inc., La Jolla, CA, USA). For combinational treatments the combination index (CI) was calculated using Calcusyn software (Biosoft, Great Shelford, Cambridge, UK) or Compusyn software (ComboSyn Inc., Biosoft; Cambridge, UK), based on the Chou and Talalay method^31^. For details see *Supplementary Information*.

### Colony-forming unit assays

To test treatment-related toxicity, CD34+ cells from healthy donors (n = 3) were exposed to the corresponding drugs or DMSO in methylcellulose medium (Methocult Express ref. 4437, StemCell Technologies SARL, Grenoble, France). Colony-forming units (CFU- granulocyte-monocyte and erythroid colonies)^32^ were scored on day 13. For details see *Supplementary Information*.

### *In vivo* studies

Female 5–6 week old NSG (NOD.Cg-*Prkdc*^*scid*^*Il2rg*^*tm1Wjl*^*/*SzJ) mice (The Jackson Laboratory, Bar Harbor, ME, USA) were injected with 5 × 10^6^ OCI-AML3 (*FLT3*^WT/WT^) cells. Mice were treated daily with vehicle (10% DMSO, n = 7), trametinib (0.5 mg/kg, n = 7)^24^, midostaurin (50 mg/kg, n = 7)^33^ or the combination of both (n = 6) over 14 continuous days, and sacrificed when AML symptoms appeared along the experimental period of 57 days. Experiments involving animals were conducted at the Centro Nacional de Investigaciones Oncológicas (CNIO) in accordance with National and International Guidelines for Animal Care. The study protocol was approved by the Institutional Animal Care and Use Committee of the Comunidad de Madrid on April 18^th^ 2017. For details see *Supplementary Information*.

### Statistical analysis

Data are presented as the mean and standard error of the mean (sem) or median values for survival analysis. Comparisons of means of variables between different groups were performed using the parametric Student’s *t* test (two-sided) when the population followed a Gaussian distribution, or the non-parametric Mann-Whitney test when they did not. Overall survival curves were performed using the Kaplan-Meier estimation, and the Mantel-Cox test was used for comparisons between groups. Univariable Cox proportional hazard ratio (HR) models were applied to investigate the influence of treatment in overall survival. A *P* ≤ 0.05 was considered significant. All statistical analyses were performed with GraphPad Prism software and SPSS v23 statistics software (North Castle, NY, USA).

## Results

### ERK1/2 pathway is activated in TKI-treated *FLT3*-mutated patients

Between 2008 and 2016, five patients with AML with mutated *FLT3* received TKI treatment in our hospital and, after a few months, three of them developed resistance (patients #1, #2, and #3, see Table 1). Patient #1 was diagnosed with AML, French-American-British (FAB) classification M1, presenting a point mutation in the JMD of *FLT3* (L576P). Whole exome sequencing was used to confirm the absence of mutations in genes related to the main FLT3 downstream signaling pathways (ERK1/2, PI3K/AKT, and JAK/STAT). The patient was included in the PANOBINODARA clinical trial (NCT00840346), but relapsed after some months. Compassionate use of sorafenib was administered after informed consent and institutional review board approval. Despite a good initial response, the disease progressed and the patient died 33 months after AML diagnosis.

**Figure 1:**
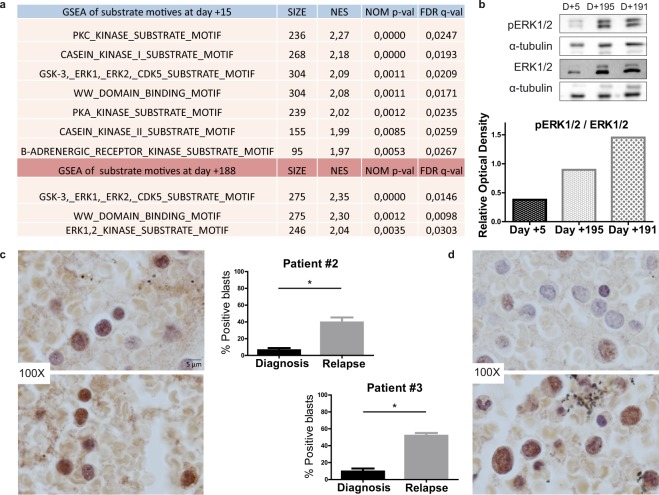
ERK1/2 is activated after continued TKI treatment in *FLT3*-mutated AML. (**a**) Venn diagram of enriched substrate motifs at the two points of treatment in patient #1. (**b**) Western blot of phospho-ERK1/2 levels on different days (**D**) of treatment in patient #1. (**c**) Immunohistochemistry analysis of phospho-ERK1/2 levels of patient #2 at diagnosis (above) and relapse after TKI treatment (below). (**d**) Immunohistochemistry analysis of phospho-ERK1/2 levels in patient #3 at diagnosis (above) and relapse after TKI treatment (below). *P ≤ 0.05. Scale bar: 5 µm.

Two peripheral blood mononuclear cell (PBMC) samples from patient #1 (day +15 and +188 of sorafenib treatment) were analyzed by LC-MS/MS after phosphopeptide enrichment. We performed Kinase Set Enrichment Analysis of substrate motifs using MaxQuant software. The library was used to predict the putative kinase activities responsible for the input set of identified phosphosites. The analysis revealed seven enriched substrate motifs at the beginning of the treatment (day +15) and three enriched substrate motifs after sustained TKI treatment (day +188). ERK1/2 kinase substrate motif was the only motif identified at day 188 but not at day +15, indicating increased ERK1/2 activity after persistent TKI treatment (Fig. 1a).

We then analyzed phospho-ERK1/2 levels of three different PBMC samples from patient #1 (day +5, day +191, and day +195 of sorafenib treatment) by western blotting, finding that ERK phosphorylation levels were significantly higher after 6 months of sorafenib treatment (Fig. 1b).

To corroborate this potential mechanism of TKI resistance, we analyzed the levels of phospho-ERK1/2 levels in the other two TKI-treated patients. Patient #2 was also diagnosed with AML-M1, with a point mutation in the JMD of *FLT3* (L576P). After several therapeutic lines, compassionate use of sorafenib was administered after informed consent and institutional review board approval, and the patient achieved a hematological response for 5 months. Thereafter, bone marrow analysis revealed the recurrence of blasts, and the patient relapsed and died 22 months after AML diagnosis. Patient #3 was diagnosed with AML-M2 with *FLT3*-ITD mutations and was recruited to a midostaurin clinical trial (NCT00651261). But despite having an initial good response, the patient relapsed 5 months later and died 8 months after AML diagnosis.

Paraffin-embedded bone marrow clots at diagnosis and relapse from patients #2 and #3 were analyzed by immunohistochemistry for phospho-ERK1/2 expression. The percentage of stained blasts was calculated before TKI treatment and at relapse. Significant differences were observed in both patients, with an increase in pERK1/2-positive blasts after TKI treatment. Higher levels of the phosphorylated form were recorded in patient #2 (Fig. 1c) and patient #3 (Fig. 1d) at relapse. These findings corroborate those observed in patient #1 by phosphoproteomics.

### ERK1/2 pathway is activated after TKI resistance *in vitro*

**Figure 2:**
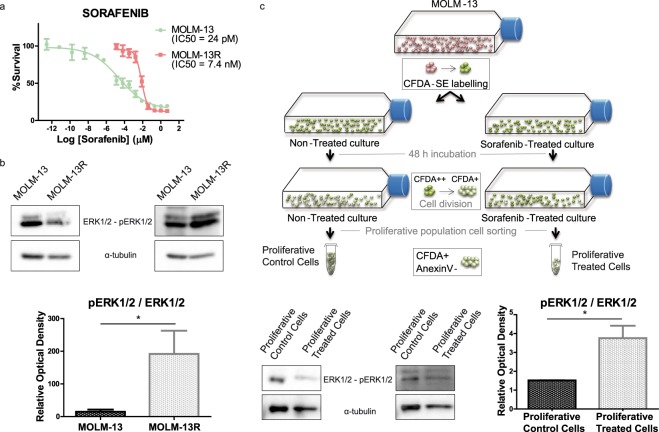
ERK1/2 pathway is activated after TKI-resistance *in vitro*. (**a**) Dose-response curve of sensitive or resistant (R) MOLM-13 cells. The IC_50_ value for sorafenib was almost 300 times higher than the control IC_50_ value in MOLM-13R cells. (**b**) pERK/ERK protein levels measured by western blotting in sensitive and resistant MOLM-13 cultures. The intensity of each band was normalized to the corresponding α-tubulin value. *P ≤ 0.05. (**c**) pERK/ERK levels of proliferative MOLM-13 cultures stained with CFDA-SE and sorted after 48 h of sorafenib treatment. pERK and ERK levels were analyzed by western blotting and normalized to α-tubulin. *P ≤ 0.05.

Given these findings, we next tested whether ERK phosphorylation could be induced by the TKI treatment *in vitro*. To study resistance mechanisms, we used the MOLM-13 cell line that expresses *FLT3*-WT and *FLT3*-ITD. We observed that sustained treatment with increasing doses of sorafenib resulted in acquired resistance. This resistant cell line (MOLM-13R) was approximately 300-times less sensitive to sorafenib than the sensitive MOLM-13 cell line, which presented an IC_50_ value in the picomolar range (IC_50_ values are shown in Fig. 2a). We next studied the effect of the TKI midostaurin and observed cross-resistance in the MOLM-13R cell line but effectiveness in sensitive MOLM-13 cells, with an IC_50_ value in the nanomolar range (see *Supplementary Information*, Supplementary Fig. S1).

Western blot analysis revealed that the MOLM-13R cell line showed significantly higher expression of pERK1/2 than parental MOLM-13 cells (Fig. 2b), similar to our observations in *ex vivo* patient cells (Fig. 1b). To validate ERK activation as a common mechanism of TKI resistance, we analyzed the resistant population selected after 48 h of sorafenib treatment *in vitro* using the same cell line background. Live treated proliferative cells (CFDA+ and Annexin V-), separated by sorting (see *Supplementary Information*, Supplementary Fig. S2), exhibited higher ERK1/2 phosphorylation levels than DMSO-treated control (Fig. 2c).

### Trametinib is effective and has a synergistic effect with TKI in MOLM-13 and MOLM-13R cells

**Figure 3:**
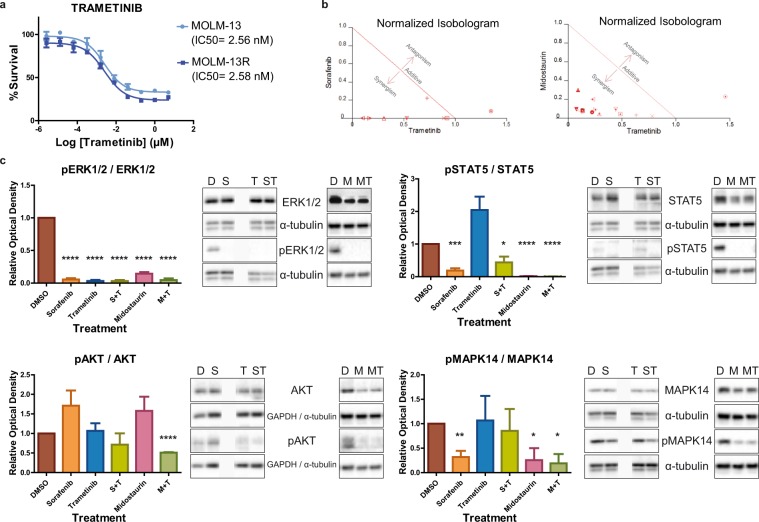
Trametinib effectively inhibits MOLM-13 and MOLM-13R cells and synergizes with sorafenib or midostaurin in MOLM-13 cells. (**a**) Dose-response curve of trametinib in sensitive and resistant (R) MOLM-13 cells. (**b**) Normalized isobolograms for trametinib in combination with the TKIs sorafenib and midostaurin in MOLM-13 cells. (**c**) The levels of ERK1/2, STAT5, AKT, and MAPK14 and their phosphorylated forms were analyzed by western blotting in TKI-sensitive MOLM-13 cultures after monotherapy or combined drug treatments (200 nM of each treatment for 3 hours). Representative blots of three independent experiments, yielding equivalent results, are shown. *P ≤ 0.05, **P ≤ 0.01, ***P ≤ 0.001, ****P ≤ 0.0001.

The apparent upregulation of the ERK1/2 pathway in TKI-resistant AML prompted us to hypothesize that patients with AML might benefit from ERK inhibition. We thus tested whether the MEK inhibitor trametinib is effective against both sensitive and TKI-resistant MOLM-13 cells. Both cell lines were equally sensitive to trametinib, which efficiently inhibited cell growth *in vitro* in the low nanomolar range (IC_50_ MOLM-13 = 2.56 nM, IC_50_ MOLM-13R = 2.58 nM, Fig. 3a). These results indicate that there was no cross-resistance to trametinib in the tested cell lines, and suggest that trametinib may be beneficial in the context of TKI-resistant *FLT3*-mutated AML.

As trametinib in combination with TKIs might prevent the development of resistance, we next determined the effects of combining trametinib with sorafenib or midostaurin. Trametinib doses from 20 nM to 2.5 nM were tested with different doses of sorafenib (5 nM to 5 fM), and midostaurin (40 nM to 1.25 nM) to determine the CI. To be more restrictive, we only considered strong synergism when the CI was ≤0.5. After 48 h of simultaneous treatment, strong synergistic effects were observed for both combinations (Fig. 3b), indicating that the combination of trametinib with either of the tested TKIs potentiated the inhibition of MOLM-13 cell growth. The effects of combining trametinib (200 nM to 12.8 pM) with midostaurin (5 μM to 64 pM) were also evaluated in MOLM-13R cells. Strong synergistic effects were observed in the TKI-resistant MOLM13-R cell culture (*Supplementary Information*, Supplementary Fig. S3a).

### Midostaurin + trametinib is the most effective combination of drugs *in vitro*

To explore the effects of the different drugs on FLT3 downstream signaling pathways, we tested drugs *in vitro* as monotherapy or in combination in MOLM-13 cells. Because a rebound phenomenon has been described after TKI treatment, with profound early inhibition followed by increasing ERK phosphorylation after incubation periods of 8–24 hours^22^ we chose a shorter time window (3 hours).

Western blot analysis indicated a complete loss of pERK signaling after treatment with sorafenib, midostaurin, trametinib, and their combinations (Fig. 3c). Similarly, phospho-STAT5 levels were significantly decreased relative to the DMSO control after sorafenib or midostaurin treatment (Fig. 3c). By contrast, trametinib treatment led to a non-significant increase in pSTAT5 levels, but its combination with sorafenib or midostaurin reversed this trend. Analysis of AKT phosphorylation showed that whereas trametinib treatment had no effect on pAKT levels, its combination with the TKIs resulted in diminished pAKT levels, which was significant for the trametinib plus midostaurin combination. The same effects were observed after sustained TKI treatment, in TKI-resistant MOLM13-R cells (*Supplementary Information*, Supplementary Fig. S3b).

Finally, we also analyzed MAPK14 activation because of its role in the development of sorafenib resistance in other types of tumors^18^. Similar to the results for AKT phosphorylation, trametinib had no effect on pMAPK14 levels, whereas sorafenib and midostaurin diminished them (Fig. 3c). Midostaurin plus trametinib was the only combination that notably decreased pMAPK14 levels. Thus, trametinib plus midostaurin seems to be the most effective combination, as it was capable of inhibiting the four analyzed pathways.

### Midostaurin + trametinib exhibit synergy *in vitro* and *ex vivo* on *FLT3* wild-type AML

Because midostaurin has activity against both mutated and wild-type *FLT3*, and the receptor is activated in almost all types of AML, its combination with trametinib might be a promising treatment for AML. We first tested whether ERK phosphorylation could also be induced by TKI treatment *in vitro* in the *FLT3*-WT cell line OCI-AML3. To do this, we treated OCI-AML3 cells with increasing doses of midostaurin. Western blot analysis revealed that this new resistant cell line showed higher expression of pERK1/2 than parental cells (*Supplementary Information*, Supplementary Fig. S4a).

**Figure 4:**
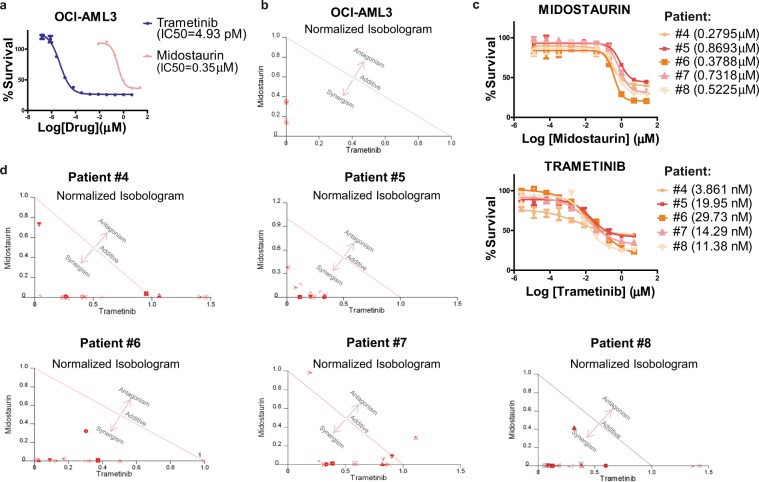
Midostaurin plus trametinib exert synergistic cytotoxicity in *FLT3*-WT AML samples *in vitro* and *ex vivo*. (**a**) Dose-response curve of trametinib and midostaurin in an *FLT3*-WT culture (OCI-AML3). (**b**) Normalized isobolograms for trametinib in combination with the TKI midostaurin in *FLT3*-WT OCI-AML3 cells. (**c**) Dose-response curve of midostaurin and trametinib in five *ex vivo FLT3*-WT AML samples. (**d**) Normalized isobolograms for trametinib in combination with the TKI midostaurin in five *ex vivo FLT3*-wildtype AML samples.

Both drugs were effective as monotherapy (Fig. 4a) and in combination (strong synergy, CI ≤ 0.5) (Fig. 4b) in the *FLT3*-WT cell line (OCI-AML3). Sorafenib was also effective as monotherapy (data not shown), but its combination with trametinib did not produce strong synergy (CI ≥ 0.5).

To study the possible role of MEK inhibition in the prevention of the emergence of acquired TKI resistance in *FLT3-*WT background, we examined the resistant population after 48 h of treatment of OCI-AML3 cells with midostaurin, trametinib or their combination. Live treated proliferative cells (CFDA+ and Annexin V-) showed decreased survival after the combination *versus* midostaurin alone (*Supplementary Information*, Supplementary Fig. S4b).

The enhanced antileukemic activity of trametinib plus midostaurin suggests that simultaneous inhibition of the ERK1/2 pathway and FLT3 signaling (wild-type or mutated) might be an effective treatment strategy for AML patients. We thus tested this combination *ex vivo* in cells from five patients (patient #4 to patient #8, all with *FLT3*-WT, see Table 1). Cytotoxicity and strong synergy (CI ≤ 0.5) were observed in each case; IC_50_ values for midostaurin were 0.28–0.87 µM, and for trametinib 3.86–29.73 nM (Fig. 4c), which are in line with the IC_50_ values obtained *in vitro*. Also, strong synergy effects (CI ≤ 0.5) were observed in the majority of tested combinations (Fig. 4d). Overall, these results support the use of midostaurin + trametinib in *FLT3*-WT AML cells.

### Midostaurin + trametinib is safe *ex vivo* in healthy CD34+ cells

**Figure 5:**
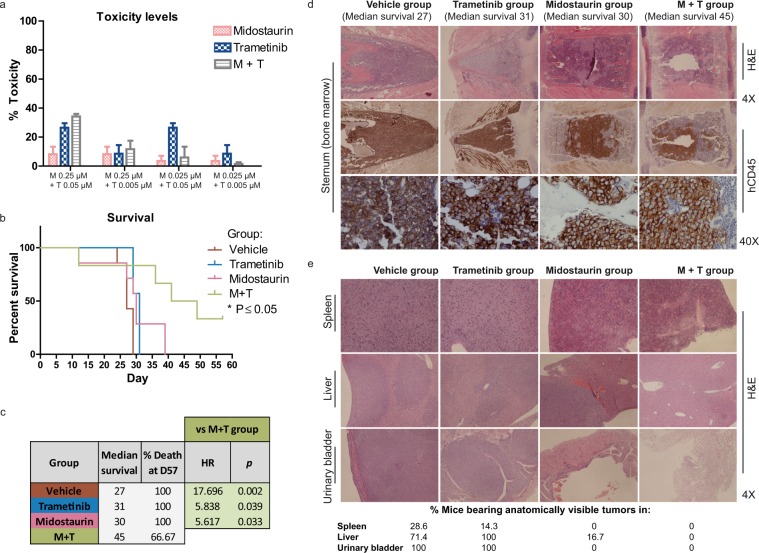
Midostaurin plus trametinib is safe in healthy donor cells and significantly improves survival over monotherapy *in vivo* (**a**) Toxicity levels for both colony populations (granulocyte-monocyte or erythroid CFU) at indicated doses after 13 days of growth in methylcellulose medium. Data are expressed as percentage of toxicity relative to DMSO control (n = 3). (**b**) Survival curves of vehicle, trametinib, midostaurin and combination groups from *in vivo* studies. Statistically significant differences between combination group and vehicle (*P = 0.0134), combination and midostaurin group (*P = 0.0295), and combination and trametinib group (*P = 0.0153) were observed, with the combination treatment significantly improving survival. (**c**) Table showing median survival in days, % death at day 57 and hazard ratio and p-values from each group of treatment. (**d**) Representative hematoxylin and eosin (H&E) and human-CD45 stained sternum slides at 4X and 40X showing OCI-AML3 cell infiltration in bone marrow. (**e**) H&E stained slides from spleen, liver and urinary bladder showing tumor or non-tumor sections in each case. The percentage of mice bearing anatomically visible tumors from each treatment group is represented below.

To test whether the combination of midostaurin plus trametinib could affect the colony formation of granulocyte-monocyte or erythroid colonies, we tested different combinational doses within the range of combination IC_50_ values observed for leukemic AML cells. Similar doses were able to inhibit leukemic cell growth without affecting healthy progenitor cells. Only mild cytotoxicity was observed at 0.25 µM midostaurin plus 0.05 µM trametinib (Fig. 5a), and slight toxicity, if any, was observed in the remainder of the assayed combinations. We would assume that the toxicity would be the same, or lower with each drug used separately.

### Midostaurin + trametinib significantly improves survival over monotherapy in an *in vivo* AML model

Finally, to verify the efficacy and safety of the combination, we performed an *in vivo* study with OCI-AML3 cells injected into NSG mice. We found statistically significant survival differences between the midostaurin + trametinib combination (M + T) and the vehicle (V) group (P = 0.0134), the M + T and the T group (P = 0.0153), and the M + T and the M group (P = 0.0295), with the combination treatment significantly improving survival rates (Fig. 5b). Median survival in days was V (27), T (31), M (30) and M + T (45) and the percentage of death at the end of the experiment was 66.7% for M + T and 100% for the other groups (Fig. 5c). We then compared the risk of death from each treatment group *versus* combination group based on hazard ratio analysis: vehicle group [HR, 17.696; 95%CI, 2.965–105.613; P = 0.002], trametinib group [HR, 5.838; 95%CI, 1.090–31.255; P = 0.039] and midostaurin group [HR, 5.617; 95%CI, 1.150–27.437; P = 0.033]. These results highlight the efficacy of the combination of trametinib plus midostaurin *in vivo*.

Histopathology examination of hematoxylin and eosin (H&E)- and human-CD45-stained bone marrow samples from mice showed proliferation of OCI-AML3 cells *in vivo* in all groups at necropsy (Fig. 5d). Remarkably, at the end of the experiment, two of the six mice in the combination treatment group did not show apparent leukemia symptoms, and one mouse had no infiltration of AML blasts in bone marrow, demonstrating that the combination controlled the progression of the disease for a longer period of time than the other groups. Histological examination of H&E-stained slides from spleen, liver and urinary bladder revealed some extramedullary tumors in vehicle, trametinib and midostaurin groups, but none were found in the combination treatment group (Fig. 5e). The percentage of mice bearing anatomically visible tumors is represented in Fig. 5e.

Taken together, the present data show that the combination of trametinib plus midostaurin can efficiently improve survival and control the progression of the disease for a longer period of time than monotherapy. These data also support the applicability of the combination in the context of AML *FLT3* wildtype.

## Discussion

Distinct mutations in *FLT3* lead to the constitutive activation of the receptor and its downstream pathways^3–6,17,34–36^. Several TKIs that achieve sustained *in vivo* inhibition of FLT3 have exhibited highly promising activity in early clinical studies^10,34^; however, none of them are able to ensure remission of AML as a single treatment^9^, primarily because of secondary resistance. Among the possible mechanisms for these failures is the existence of independent alternative survival pathways, such as casein kinase 2 alpha, CD47, CD123, PIM, PI3K/AKT/mTORC, JAK/STAT, and MAPK^1,3,16–20^. In the present study, we demonstrate that ERK activation is a common mechanism of TKI resistance *in vitro* and *ex vivo*. We detected ERK activation in an *FLT3*-mutated cell culture using two different means of resistance selection. Also, PBMCs from different patients harboring *FLT3* mutations showed sustained ERK activation after the development of TKI resistance, as demonstrated by phosphoproteomics, western blotting, and immunohistochemistry, with increased levels of pERK observed in the cytoplasm and nuclei of infiltrated blasts. pERK distribution is critical for substrate targeting in the nucleus (e.g., c-Fos and c-Myc) and the cytosol (e.g., ribosomal S6 kinases), although the mechanisms regulating this subcellular localization are unclear^37^. ERK activation has been previously suggested as a mechanism of TKI resistance, directly or indirectly, by the activation of upstream regulatory pathways^3,17,22,38,39^. Because of these results, we hypothesized that ERK inhibition, *via* its regulatory molecule MEK, might overcome TKI resistance, as suggested in other studies^21,22,40^. For example, Bruner *et al*.^22^, propose this combination after studying the incomplete response to TKI therapy in *FLT3*-ITD AML cells. According to these suggestions, we characterized the effect of the MEK inhibitor trametinib and its combination with the two TKIs sorafenib and midostaurin *in vitro*.

We demonstrate *in vitro* the potential use of trametinib in the context of *FLT3*-mutated AML, even in a background of TKI resistance. Indeed, previous studies have demonstrated the efficacy of trametinib in *RAS*-mutant AML^24,25^.

The activity of trametinib against TKI-resistant blasts suggests its possible use in combination with TKI to overcome resistance development. We therefore examined for synergism between trametinib and other TKIs, such as sorafenib, and midostaurin in order to look for the most beneficial combination. Of note, their combinations presented strong synergy (CI ≤ 0.5). Our *in vitro* results suggest that sorafenib is the most potent compound, and its combination with trametinib showed strong synergy. Indeed, a recent clinical trial^41^ has evaluated the effect of trametinib plus sorafenib in patients with advanced hepatocellular cancer (NCT02292173), showing a good safety profile. Although sorafenib has shown encouraging results in AML clinical trials^42,43^, and it has been recommended for compassionate use in *FLT3*-mutated patients, it has not yet been approved. Whereas the addition of sorafenib to standard treatment in AML has not improved overall survival, the addition of midostaurin has resulted in a significant overall survival benefit^44^ and better tolerance than sorafenib^45^. In 2017, midostaurin plus chemotherapy was approved by the FDA for use in AML. In line with these results, we also observed a strong potency of midostaurin in *FLT3*-mutated cell lines and strong synergy when midostaurin was combined with trametinib in sensitive and TKI-resistant cultures. Interestingly, this combination was the only one able to inhibit the downstream FLT3 pathways described as potential mechanisms of resistance^1,3,16,17,19,20^.

As *FLT3*-WT and *FLT3* mutants^46^ are sensitive to midostaurin, and the receptor is activated in almost all types of AML^4,9,47,48^, we would propose the extended use of its combination with trametinib, even in *FLT3*-WT patients. In this context, we demonstrate for the first time that ERK was also activated after sustained TKI treatment. Our results show that both compounds in monotherapy remain active against *FLT3*-WT *in vitro* and *ex vivo*. Interestingly, the IC_50_ obtained *in vitro* correlated with the *ex vivo* results. The combination was also synergistic *in vitro* and *ex vivo*, and showed no associated toxicity in CD34+ cells from healthy donors. Compellingly, the combination resulted in statistically significant improvements *in vivo* compared with control and single therapy groups. While the present study shows that trametinib is beneficial in the context of TKI resistance and the prevention of the emergence of resistance even in the *FLT3*-WT background, further studies will be needed to explore other potential pathways involved in dual resistance, which will hopefully facilitate new prevention strategies.

The ERK1/2 pathway is a relevant resistance mechanism in patients with AML treated with TKIs. Our data provide clear evidence of the potential therapeutic benefit of the combination of the FLT3 inhibitor midostaurin and the MEK inhibitor trametinib, not only in *FLT3*-mutated patients as previously suggested^22^, but also in those without the mutation, which increases the range of patients that could obtain benefits from the drug combination. This combination is synergistic *in vitro*, *ex vivo*, and *in vivo*. Lastly, the combination improves the survival rate *in vivo*. Overall, these data support a clinical trial of midostaurin plus trametinib in AML.

## Conclusions

ERK1/2 activation can promote TKI resistance in AML. We demonstrate that inhibition of the ERK1/2 pathway with trametinib, along with the use of a TKI, such as midostaurin, could be a beneficial therapeutic strategy in patients with AML and mutated or non-mutated *FLT3*.

### Ethics approval and consent to participate

Studies involving human data and tissues were approved by our Institutional Review Board and all patients provided written informed consent in accordance with the Declaration of Helsinki.

Studies involving animals were conducted in accordance with National and International Guidelines for Animal Care and the study protocol was approved by the Institutional Animal Care and Use Committee of the Comunidad de Madrid on April 18th 2017.

## Supplementary information

Supplementary Information
