# Supplementary material for: MEK inhibition enhances the response to tyrosine kinase inhibitors in acute myeloid leukemia
Source: Sci Rep. 2019 Dec 9;9:18630. doi: 10.1038/s41598-019-54901-9 (PMC6901485; doi:10.1038/s41598-019-54901-9)
Supplement: Supplementary file 1 — Supplementary Information [file 41598_2019_54901_MOESM1_ESM.pdf]

SUPPLEMENTARY INFORMATION FILE

MEK inhibition enhances the response to tyrosine kinase inhibitors in acute myeloid leukemia

María Luz Morales, Alicia Arenas, Alejandra Ortiz-Ruiz, Alejandra Leivas, Inmaculada Rapado, Alba Rodriguez-Garcia, Nerea Castro, Ivana Zagorac, Miguel Quintela-Fandino, Gonzalo Gómez-López, Miguel Gallardo, Rosa Ayala, María Linares & Joaquín Martinez-Lopez

TABLE OF CONTENTS

SUPPLEMENTARY METHODS. 3

Extended cell cultures, AML and healthy primary cells and drugs 3

Extended whole exome sequencing 4

Extended LC-MS/MS analysis 4

Sample preparation for proteomic analysis 4

Phosphopeptide enrichment 5

Mass spectrometry 5

Data analysis 6

Extended Histopathology and Immunohistochemistry 6

Extended Immunoblotting assays 6

Extended Drug Sensitivity Assay 7

Extended colony-forming unit assays 8

Extended *in vivo* studies 9

SUPPLEMENTARY FIGURES 10

Supplementary Figure S1. IC50 dose-response to midostaurin in MOLM-13 and MOLM-13R 10

Supplementary Figure S2. Representative charts/plots from flow acquisition cell sorting 10

Supplementary Figure S3. The combination of midostaurin plus trametinib is synergistic and effective in MOLM-13R 11

Supplementary Figure S4. Midostaurin plus trametinib is beneficious in the context of TKI resistance in FLT3 wildtype 12

SUPPLEMENTARY TABLES 13

Supplementary Tables 11

Supplementary Table 1. Antibodies information. 13

SUPPLEMENTARY METHODS

Extended cell cultures, AML patients, healthy donors and drugs

Human MOLM-13 ($FLT3^{ITD/WT}$) and OCI-AML3 ($FLT3^{WT/WT}$) AML cell lines were obtained from the DSMZ culture collection (Braunschweig, Germany). Other mutations are available at the *Catalogue of Somatic Mutations* (COSMIC) website (COSMIC sample ID: COSS1330947 and COSS1290455 respectively). MOLM-13 cells were cultured in RPMI-1640 medium (Biowest, Nuaillé, France) supplemented with 10% fetal bovine serum (FBS) (Thermo Fisher Scientific, Waltham, MA, USA) and antibiotics (100 U/ml penicillin and 100 µg/ml streptomycin). To induce resistance to sorafenib, the parental MOLM-13 cell line was grown in culture in the presence of 5 nM sorafenib, which was gradually increased to 20 nM over 17 days to obtain MOLM-13 resistant (MOLM-13R) cells. Culture media for MOLM-13R cells were supplemented with a final concentration of 20 nM sorafenib. The OCI-AML3 cell line was cultured in IMDM medium (Biowest, Nuaillé, France) supplemented with 5% FBS. To induce resistance to midostaurin, the parental OCI-AML3 cell line was grown in culture in the presence of 0.1 µM midostaurin, which was gradually increased to 5 µM over 10 days to obtain the resistant culture (OCI-AML3R). All cell lines were grown at 37ºC in a humidified atmosphere containing 5% CO_2, and maintained by periodic subculture every 2–3 days.

For experiments of sorafenib-resistant cells enrichment, MOLM-13 sensitive cells were stained with 5,6-carboxyfluorescein diacetate succinimidyl ester (CFDA-SE) (ThermoFisher Scientific, Waltham, MA, USA) and treated with 5 µM sorafenib for 48 h. APC Annexin V (BD Bioscience, San Jose, CA, USA) staining was used to discard dead cells. Proliferative and viable cells (CFDA-SE+, Annexin V-) were sorted in a FACSAria™ Fusion sorter (BD Biosciences, San Jose, CA, USA). Three experiments were performed independently. Cell pellets were stored at -80ºC until use. For experiments of proliferative and viable cells in OCI-AML3 background, cells were treated with 25 µM midostaurin, 5 µM trametinib or their combination for 48 h after being stained with CFDA-SE.

For experiments with primary cells, bone marrow and peripheral blood samples from patients with AML diagnosed according to French-American-British (FAB) criteria at diagnosis and at different times of treatment were obtained. Bone marrow from healthy donors was aspirated after a medical interview. In both cases, mononuclear cells were separated by density

gradient centrifugation on Ficoll. The study was approved by the *Comité Ético de Investigación Clínica* of the *Instituto de Investigación Biomédica* of the *Hospital 12 de Octubre* and all patients and donors provided written informed consent in accordance with the Declaration of Helsinki.

Sorafenib and trametinib were purchased from Selleck Chemicals (Houston, TX, USA). Midostaurin was purchased from MedChemExpress (Sollentuna, Sweden). Stock solutions were dissolved in dimethyl sulfoxide (DMSO) (Sigma-Aldrich, St.Louis, MO, USA) at 10 mM and serially diluted in culture medium to obtain the final concentrations for *in vitro* and *ex vivo* experiments. All stock solutions were stored at -80ºC.

Extended whole exome sequencing

A total of 1 µg of high-quality RNA-free genomic DNA was isolated from the bone marrow of patient #1 at diagnosis. Exonic sequences were captured through probe-hybridization, amplified, and purified following the Ion TargetSeq™ Exome Enrichment manual (MAN0006730, Life Technologies S.A., Madrid, Spain). The purified DNA was then sequenced on the Ion Proton™ System (ThermoFisher Scientific, Waltham, MA, USA). Obtained sequence reads were aligned to the human genome reference sequence (hg19) in Torrent Suite 4.4.3. Point mutations and 1 base-pair indels were restricted to variants with mean allele frequency (MAF) < 0.1%, $P < 0.05$, and a minimum of 10 reads, supporting a call of at least 20 total reads. Indels were restricted to variants with MAF < 0.1%, $P < 0.05$, and a minimum of five reads supporting a call. The Ion PGM system (ThermoFisher Scientific) was used to validate the results.

Extended LC-MS/MS analysis

Phospohoproteomic experiments were conducted at the Proteomics Core Unit of the Centro Nacional de Investigaciones Oncológicas (CNIO).

Sample preparation for proteomic analysis

Cells were lysed using 7 M urea and 2 M thiourea in 100 mM Hepes pH 7.5, supplemented with 1:1000 (v/v) of benzonase (Novagen) and 1:100 (v/v) of HaltTM phosphatase and protease inhibitor cocktail 100× (ThermoFisher Scientific). Protein concentration was determined using the Pierce® 660nm Protein Assay (Bio-Rad Laboratories, Hercules, CA, USA) using BSA as standard. Samples (250 µg) were digested using the standard FASP protocol.

Briefly, proteins were reduced (15 mM TCEP, 30 min, room temperature), alkylated (50 mM CAA, 20 min in the dark, room temperature) and sequentially digested with Lys-C (Wako) (protein:enzyme ratio 1:50, overnight at room temperature) and trypsin (Promega) (protein:enzyme ratio 1:100, 6 h at 37 °C). Resulting peptides were desalted using C18 stage-tips.

Phosphopeptide enrichment

Phosphopeptides were enriched using home-made TiO2 micro-columns. Briefly, peptides were resuspended in 6% trifluoroacetate (TFA) and 80% acetonitrile and incubated for 20 min with TiO2 beads (10 µm particle size) (GL Science Inc., Tokyo, Japan) using a sample:TiO2 ratio of 1:2. Prior to incubation, TiO2 beads were pre-conditioned with a solution of 20 mg/mL 2,5-dihydrobenzoic acid (DHB) in 80% acetonitrile, 6% TFA, for 20 min. Then, beads were sequentially washed with 100 µL of 6% TFA and 10% acetonitrile, 100 µL of 6% TFA and 100 µL of 40% acetonitrile and 6% TFA and 60% acetonitrile. Finally, phosphopeptides were eluted first with 20 µL of 5% ammonium hydoroxide and then with 20 µL 5% ammonium hydoroxide in 10% acetonitrile in the same vial.

Mass spectrometry

LC-MS/MS was done by coupling a nanoLC-Ultra 1D+ system (Eksigent) to an LTQ Orbitrap Velos mass spectrometer (ThermoFisher Scientific) *via* a Nanospray Flex source (ThermoFisher Scientific). Peptides were loaded into a trap column (NS-MP-10 BioSphere C18 5 µm, 20 mm length, Nanoseparations) for 10 min at a flow rate of 2.5 µl/min in 0.1% FA. Then, peptides were transferred to an analytical column (ReproSil Pur C18-AQ 1.9 µm, 400 mm length and 0.075 mm ID) and separated using a 129 min effective linear gradient (buffer A: 4% acetonitrile, 0.1% FA; buffer B: 100% acetonitrile, 0.1% FA) at a flow rate of 300 nL/min. The gradient used was: 0–2 min 6% B, 2–133 min 30% B, 133–133.5 min 98% B, 133.5–143.5 min 98% B and 143.5–150 min 2% B. The peptides were electrosprayed (1.4 kV) into the mass spectrometer with a PicoTip emitter (360/20 Tube OD/ID µm, tip ID 10 µm) (New Objective, Woburn MA, USA), a heated capillary temperature of 325°C and S-Lens RF level of 60%. The mass spectrometer was operated in a data-dependent mode, with an automatic switch between MS and MS/MS scans using a top 20 method (threshold signal ≥ 800 counts and dynamic exclusion of 45 sec). MS spectra (350-1500 m/z) were acquired in the Orbitrap with a resolution of 60,000 FWHM (400 m/z). Peptides were isolated using a 2 Th window and fragmented using collision induced dissociation with linear ion trap read out at a NCE of 38%

(0.25 Q-value and 10 ms activation time). The ion target values were 1E6 for MS (500 ms max injection time) and 5000 for MS/MS (100 ms max injection time).

Data analysis

Raw files were processed with MaxQuant (v 1.5.0.12) using the standard settings against a human protein database (UniProtKB/Swiss-Prot, December 2013, 20,584 sequences) supplemented with contaminants. Label-free quantification was done with match between runs (match window of 0.7 min and alignment window of 20 min). Carbamidomethylation of cysteines was set as a fixed modification whereas oxidation of methionines, protein N-term acetylation and phosphorylation of serines, threonines and tyrosines were set as variable modifications. Minimal peptide length was set to 7 amino acids and a maximum of two tryptic missed-cleavages were allowed. Results were filtered at 0.01 false discovery rate (peptide and protein level). Subsequently, the "phospho(STY)sites.txt" file was loaded in Perseus (v1.5.1.6) for further analysis.

Extended histopathology and immunohistochemistry

Paraffin embedding and sectioning of the different tissues (bone marrow clots, sternum, spleen, liver and urinary bladder) from patients with AML or mice were performed according to standard protocols. For phospho-ERK1/2 and human CD45 staining, formalin-fixed paraffin-embedded sections were deparaffinized and incubated overnight at 4ºC with a phospho-ERK1/2 (ref. 4370) or CD45 (ref. 13917) antibody (both from Cell Signaling Technology Inc., Danvers, MA, USA), followed by signal detection with a peroxidase-conjugated secondary antibody (EnVision+ Dual Link, ref. K4063, Agilent, Santa Clara, CA, USA for phosphor-ERK1/2 detection and anti-rabbit IgG, ref. 7074, Cell Signaling Technology for human CD45 detection) and the DAB substrate kit (ab94665, Abcam, Cambridge, UK). Slides were counterstained with Carazzi's hematoxylin solution (PanReac AppliChem, Ottoweg, Darmstadt, Germany) before mounting in DPX Mountant (Sigma-Aldrich). Slides images were taken with a digital camera connected to the 80i microscope, both from Nikon (Izasa S.A., Barcelona, Spain).

Extended immunoblotting assays

Cultured cells after treatment (200 nM of each treatment for 3 hours; or DMSO in control sample) or mononuclear cells were washed with ice-cold phosphate buffered saline (PBS) (Lonza, Basel, Switzerland) and pellets were collected. Cell pellets were treated with a cold

lysis buffer mixture (50 mM Tris, 150 mM NaCl, 1% Triton, 0.5% sodium deoxycholate, 0.1% SDS, protease and phosphatase inhibitors, 100 U/ml DNase I, distilled H_2O) for 30 minutes on ice and, subsequently centrifuged at 14000 × g for 5 minutes at 4ºC. Supernatants were collected and stored at -80ºC until analysis. Protein concentrations were determined using the Quick Start™ Bradford 1× Dye Reagent (Bio-Rad Laboratories), following the manufacturer's instructions, and read with the Epoch microplate reader running Gen5 software (both from BioTek, Winooski, VT, USA).

Whole cell lysates were separated by SDS-PAGE and then transferred to polyvinylidene difluoride Immun-Blot® membranes (Bio-Rad Laboratories). Proteins were detected after overnight incubation at 4ºC with primary antibodies (see Supplementary Table 1). Antibodies against α-tubulin, GAPDH or ERK1/2, AKT, STAT5, MAPK14 and their phosphorylated forms were used. Then, secondary antibodies (see Supplementary Table 1) conjugated with horseradish peroxidase were incubated for 1 hour at room temperature. Bound antibodies were detected using the Prime Western Blotting Detection Reagent Amersham™ (GE Healthcare, Little Chalfont, UK) and the ChemiDoc™ MP Imaging System (Bio-Rad Laboratories). Protein expression (densitometry analysis) was determined using ImageLab 5.0 software (Bio-Rad Laboratories) and corrected for housekeeping protein expression. Finally, expression levels were normalized to control samples and statistical analyses were performed.

Extended drug sensitivity assay

Growth analyses were performed by seeding cells into 96-microwell plates at $2×10^5$ cells/ml or $3×10^5$ cells/ml (primary cells and MOLM-13 or OCI-AML3 cell lines, respectively). Cell viability after monotherapy and combination treatments (trametinib in combination with the three different TKIs) was determined after 48 hours or 72 hours (in cell lines or primary cells respectively) of exposure to drugs or DMSO alone using the Cell Counting Kit-8 reagent from Sigma-Aldrich, according to the manufacturer's protocol, and measured with the Epoch microplate reader running Gen5 software.

For monotherapy treatments *in vitro*, cell survival percentages from 3–6 independent experiments were calculated and normalized to controls. The half maximal inhibitory concentration (IC_{50}) values were determined according to a nonlinear program by GraphPad

Prism 5.01 (La Jolla, CA, USA) and dose-response graphics were represented as the mean survival ± standard error of the mean (sem).

For combinational treatments *in vitro*, drug effects from 3 independent experiments were tested and the combination index (CI) was calculated. Normalized isobolograms were represented using Calcusyn software (Biosoft, Great Shelford, Cambridge, UK).

For monotherapy and combination treatments *ex vivo*, the average percentage of blasts was around 70% in patients, so we can almost certainly exclude that the toxicity of the drug combination was due to the effects on other non-leukemic mononuclear cells. Bone marrow mononuclear cells were cultured in StemSpan™ SFEM medium (StemCell Technologies SARL, Grenoble, France) containing 20% FBS (ThermoFisher Scientific), Human Recombinant Stem Cell Factor (100 ng/ml) (StemCell Technologies), Human Recombinant Interleukin-3 (20 ng/ml) (StemCell Technologies), Human Interleukin-6 (20 ng/ml) (Miltenyi Biotec S.L., Pozuelo de Alarcón, Madrid, Spain), Human Recombinant FLT3-Ligand (100 ng/ml) (PeproTech EC Ltd., London, UK), and the corresponding drug therapies. Data were collected from 3 technical replicates for each patient and the applied analysis was the same as for the *in vitro* experiments.

Extended colony-forming unit assays

To test for treatment-related toxicity, colony-forming assays in CD34+ control cells were performed. CD34+ cells were isolated from mononuclear cells with the MACs CD34 MicroBead Kit (Miltenyi Biotec S.L.) according to the manufacturer's instructions. In total, 2×10^3 CD34+ cells were resuspended in 200 µl of IMDM medium (Biowest, Nuaillé, France) supplemented with 20% FBS (ThermoFisher Scientific) and were then submerged into 3.8 ml of methylcellulose (Methocult Express 4437, StemCell Technologies) with different concentrations of the corresponding drugs (midostaurin, trametinib or their combination) or DMSO as control. Doses ranging from 2.5 µM to 0.025 µM were assayed for midostaurin in monotherapy, whereas trametinib monotherapy doses ranged from 5 µM to 0.005 µM. For combination treatment, doses of 0.25 µM and 0.025 µM of midostaurin were combined with doses of 5 µM, 0.5 µM, 0.05 µM and 0.005 µM of trametinib. Cells were plated in 35-mm Petri dishes in triplicates of 1.1 ml and incubated at 37ºC in a humidified atmosphere containing 5% CO_2. Colony-forming units (CFU-granulocyte-monocyte and erythroid colonies) were

scored at day 13 using a stereomicroscope Olympus VMZ 1 x – 4 x (Olympus Iberia S.A.U., Barcelona, Spain).

Extended *in vivo* studies

Female JAX^{TM} NSG mice (NOD.Cg-*Prkdc*^{scid}*Il2rg*^{tm1Wjl}/SzJ) from The Jackson Laboratory (Bar Harbor, ME, USA) were used at the age of 5–6 weeks. Standard rodent diet and water were available *ad libitum* throughout the study. Mice were housed seven per cage, each cage being a treatment group. Mice were injected with 5×10^6 OCI-AML3 cells resuspended in PBS by tail injection. Three days after injection, mice were treated with vehicle (10% DMSO, n=7), trametinib (0.5 mg/kg, n=7), midostaurin (50 mg/kg, n=7) or the combination of both (trametinib and midostaurin at the same single doses, n=6) per day by intraperitoneal injection during 14 days. Mice were monitored and sacrificed when AML symptoms or humane endpoint appeared (10% weight loss, kyphosis, paralysis, pain parameters, etc.) during the period of 57 days of the experiment. At necropsy, spleen and liver were weighed, and organs of interest were collected and embedded in paraffin blocks for further studies. All experiments involving animals were conducted at the Centro Nacional de Investigaciones Oncológicas (CNIO) in accordance with National and International Guidelines for Animal Care. The study protocol was approved by the Institutional Animal Care and Use Committee of the Comunidad de Madrid on April 18th 2017.

SUPPLEMENTARY FIGURES

Supplementary Figure S1

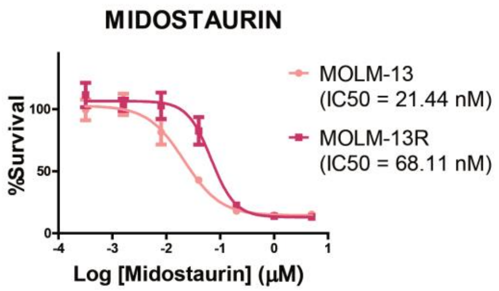

Supplementary Figure S1. IC50 dose-response to midostaurin in MOLM-13 and MOLM-13R. IC50 dose-response to midostaurin of sensitive or resistant MOLM-13 cultures (MOLM-13 and MOLM-13R, respectively).

Supplementary Figure S2

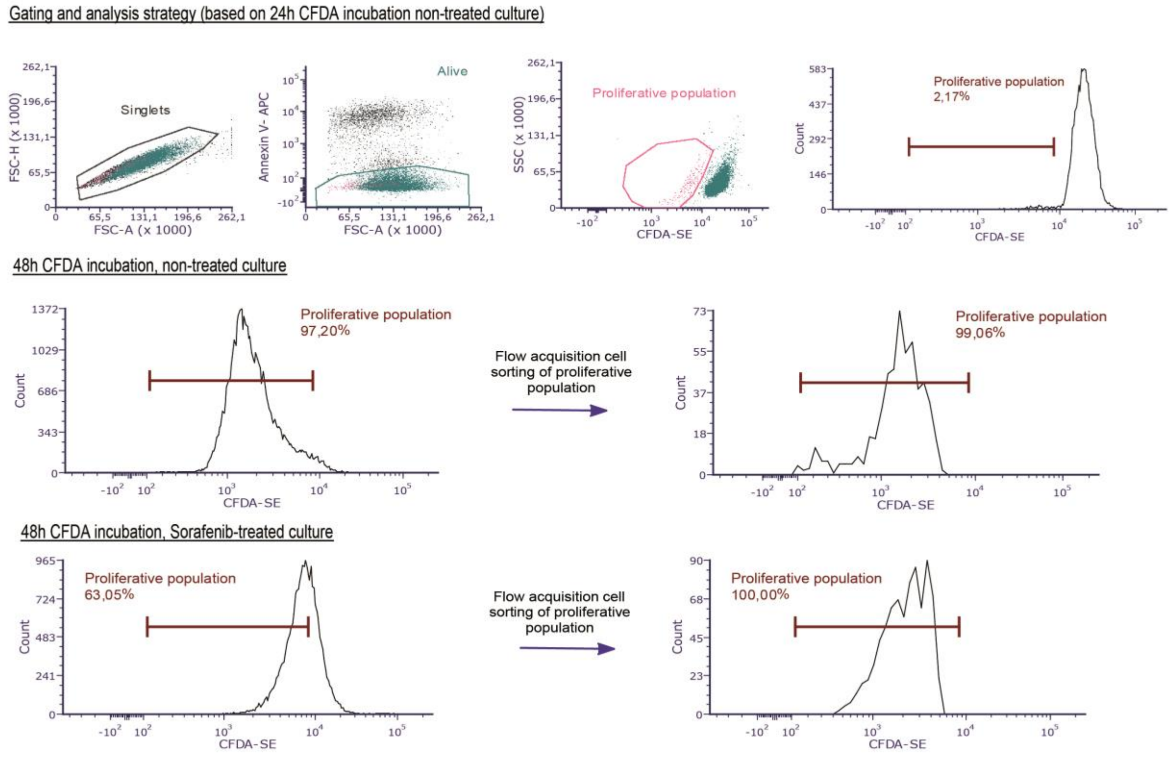

Supplementary Figure S2. Representative charts/plots from flow acquisition cell sorting. Gating strategy was based on singlets and dead cells exclusion. When possible, a minimum of 10000 cells from the population of interest were analyzed.

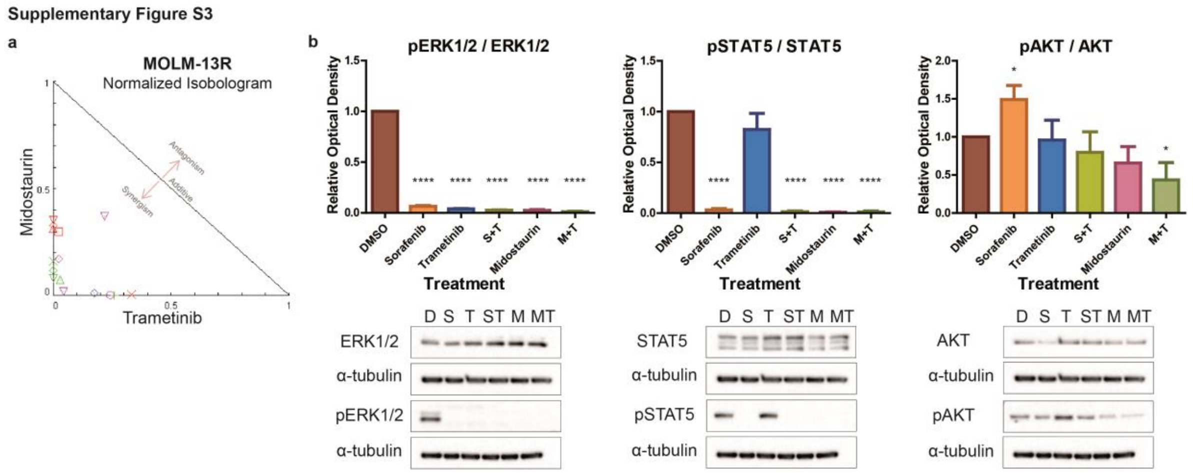

Supplementary Figure S3. The combination of midostaurin plus trametinib is synergistic and effective in MOLM-13R.

(a) Normalized isobologram for midostaurin plus trametinib combination in MOLM-13R. (b) The levels of ERK1/2, STAT5, and AKT and their phosphorylated forms were analyzed by western blotting in TKI-resistant MOLM-13R cultures after monotherapy or combined drug treatments (200 nM of each treatment for 3 hours). Representative blots of three independent experiments, yielding equivalent results, are shown. *P ≤ 0.05, **P ≤ 0.01, ***P ≤ 0.001, ****P ≤ 0.0001.

Supplementary Figure S4

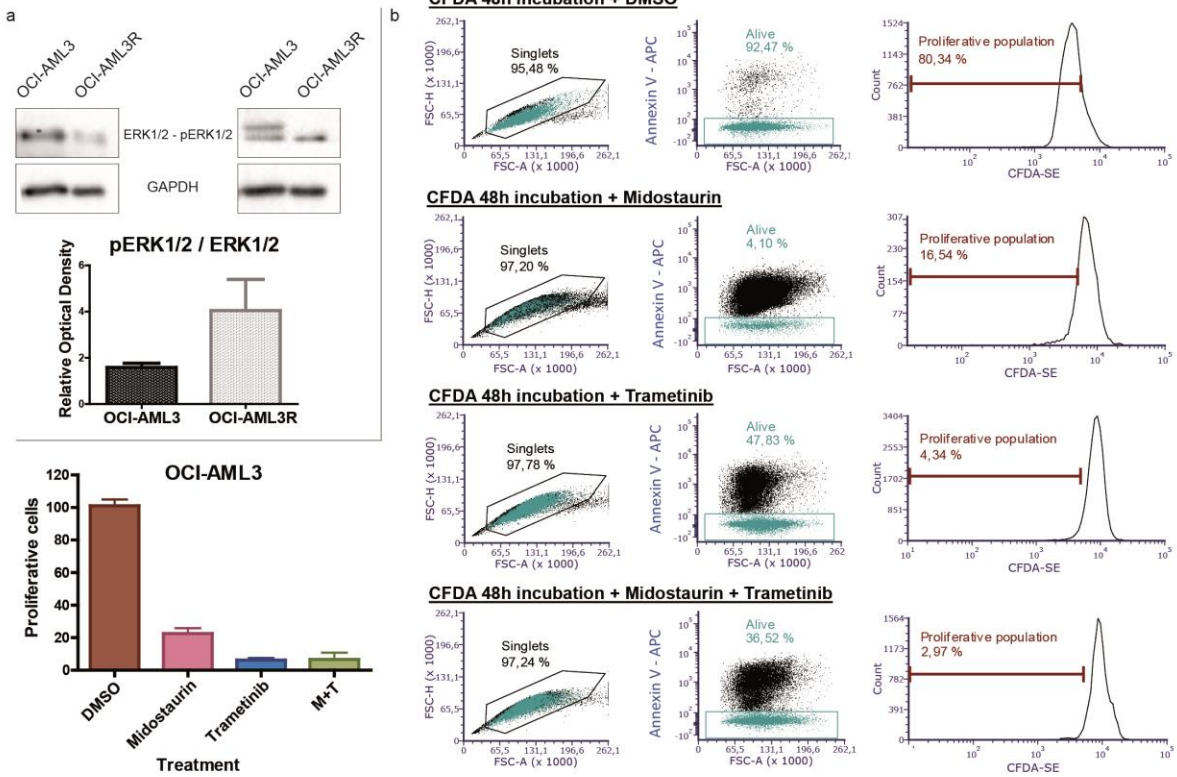

Supplementary Figure S4. Midostaurin plus trametinib is beneficious in the context of TKI resistance in *FLT3* wildtype.

(a) pERK/ERK protein levels measured by western blotting in sensitive and resistant OCI-AML3 cultures. The intensity of each band was normalized to the corresponding GAPDH value. (b) Representative charts/plots for experiments of proliferative and viable OCI-AML3 cells after midostaurin, trametinib or their combination. Gating strategy was based on singlets and dead cells exclusion. When possible, a minimum of 10000 cells from the population of interest were analyzed.

SUPPLEMENTARY TABLES

Supplementary Table 1. Antibodies information.

Name	*Reference*	*Company*	*Dilution*
**Primary antibodies**			
P44/42 MAPK (Erk1/2)	#4695	Cell Signalling TECHNOLOGY	1/1000
Phospho-p44/42 (Erk1/2)	#9106	Cell Signalling TECHNOLOGY	1/2000
Stat5	#9363	Cell Signalling TECHNOLOGY	1/1000
Phospho-Stat5	#9359	Cell Signalling TECHNOLOGY	1/100
Akt	#4691	Cell Signalling TECHNOLOGY	1/1000
Phospho-Akt	#4060	Cell Signalling TECHNOLOGY	1/2000
p38 MAPK	#9212	Cell Signalling TECHNOLOGY	1/1000
Phospho-p38 MAPK	#9215	Cell Signalling TECHNOLOGY	1/1000
Anti-α-Tubulin	AB7291	Abcam	1/10000
Anti-GAPDH	AM4300	Ambion / Invitrogen	1/10000
**Secondary Antibodies**			
Anti-rabbit IgG, HRP-linked Antibody	#7074	Cell Signalling TECHNOLOGY	1/2000
Anti-mouse IgG, HRP-linked Antibody	#7076	Cell Signalling TECHNOLOGY	1/10000
